# An Overview on Bipolar Junction Transistor as a Sensor for X-ray Beams Used in Medical Diagnosis

**DOI:** 10.3390/s22051923

**Published:** 2022-03-01

**Authors:** Luiz A. P. Santos

**Affiliations:** 1 CNEN/CRCN-NE, Recife 50740-545, Brazil; lasantos@scients.com.br; 2 SCIENTS, Igarassu 53645-337, Brazil

**Keywords:** bipolar junction transistor, X-ray, sensor

## Abstract

Although not manufactured to be used under X-ray photons, the commercial bipolar junction transistor (BJT) is an electronic device that can be used as an ionizing radiation sensor. In this article an overview on the BJT and its principle of operation were made for the purpose of better understanding how such a semiconductor device behaves when under diagnostic X-ray beam. Therefore, it addresses some topics such as the structure of the device, the bias configuration when operating in active mode, and so on. Even knowing that the most complete theory to describe the “transistor effect” is based on quantum theory (the energy band theory of solids), here it is preferable to take a simpler experimental approach to clearly understand the operation of the BJT. In electronics, the BJT is used as a current amplifier, and depending on the bias and point of view it also becomes a voltage amplifier. In the analysis of BJT under an X-ray beam, in addition to its operation as a sensor to measure the dose or some diagnostic X-ray tube parameter, it has also led to technological innovation in the technique of digital data storage based on the effect of radiation.

## 1. Introduction

This overview discusses the bipolar junction transistor (BJT) as a sensor for the X-ray beam, which is commonly used in medical diagnosis. The study becomes important for measuring the radiation dose in patients or workers exposed to X-ray beams, accurate dosimetry in phantoms, and also has great importance in the innovation of techniques and instruments for non-invasive monitoring of X-rays tube parameters used in radiology, such as kV measurement, for example. In parallel to the study of the BJT as an X-ray sensor, technological innovation has emerged for the storage of digital data based on the effect of radiation on the device, which can bring a technological breakthrough to the information technology area.

### 1.1. A Brief Background on Bipolar Junction Transistor

The BJT is an electronic device that is part of the most varied types of electronic equipment both in discrete form and the well-known integrated circuit (IC) form [1]. The bipolar transistors are also part of several types of industrial controllers, electronic systems for vehicles, airplanes, ships, linear accelerators, scientific equipment, test and measurement instruments, among others. The first commercial transistors were manufactured in large scale in the 1960s, when the first radios and TVs made with these solid state devices appeared in stores to replace electronic equipment with electronic valves [2]. At that time, the success of this innovation was absolute, since the researchers of the “transistor effect” had won a Nobel Prize in 1956 and the transistor became a milestone that changed our lives. The BJT is built with semiconductor material and the most common today is still silicon crystal doped with some types of impurities. In reality, it is not enough to have a semiconductor material to design a transistor. In fact, it is necessary to have a structure of two types of semiconductor materials (n and p) to form an npn (or pnp) structure, that is, it consists of two pn junctions. This article assumes that the reader is familiar with this semiconductor terminology, as well as the pn junction. A detail of the bipolar transistor junctions is that the material of the medium consists of a thin slice slightly doped of p-type (n-type) semiconductor sandwiched between two pieces of n-type (p-type) semiconductor. In electronics, it is said that the pnp BJT is the complementary pair (dual) of the npn BJT and vice versa. The physical principle of the transistor operation can be found in semiconductor devices and physics books [3,4,5], including some manufacturing information concerning various types of transistors. Furthermore, the reader can appreciate the quantum theory applied to the electrical conductivity of solids with equations for generation and recombination of electron-hole pairs, calculations of the electron current in the semiconductor material, and so on [5]. Also, one can find dozens of references describing on the main topics for a deeper knowledge of the bipolar transistor in the books cited above, and others [6,7,8,9]. Another semiconductor electronic device that is well-known is the field-effect transistor (FET), which was also technologically developed at about the same time as the BJT, as well as the a device called MOSFET (Metal-Oxide-Semiconductor-FET) that had its commercial explosion in the 1970s as a result of the appearance of the first logic integrated circuits and microprocessors made with them.

**Figure 1:**
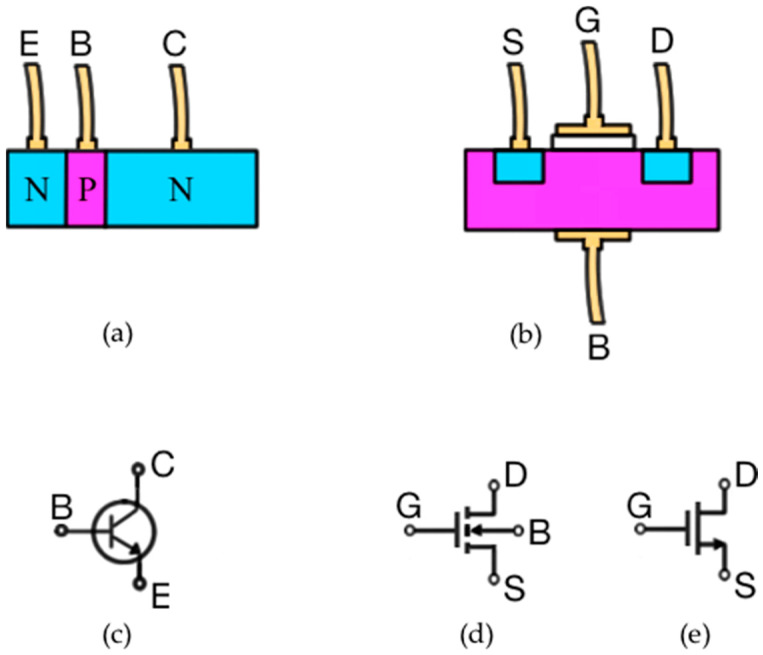
(**a**) Didactic transistor structure for a npn BJT; (**b**) Didactic structure for a n-channel MOSFET; (**c**) Symbol for a npn BJT; (**d**) A symbol for n-channel MOSFET; (**e**) Another typical symbol for n-channel MOSFET.

However, MOSFET is not the focus of this article. Therefore, for didactic purposes, it is important to compare one structure of the two main types of transistors: MOSFET and BJT. Figure 1 illustrates their respective electronic symbols and they are defined in this paper as didactic designs for each type of transistor. The three terminals of the BJT are called the emitter (E), base (B), and collector (C); and in the MOSFET the terminals are called the source (S), gate (G), drain (D), and body (B). Generally, the body and source terminals are short-circuited during MOSFET manufacturing so that there are only three terminals: S, G and D.

Note that although both semiconductor structures are npn, they are different. In the BJT the emitter is more doped than the base and geometrically smaller than the collector; the base is made very thin and also weakly doped; and there are two contributing components in the current-conduction process: electrons and holes flowing in opposite directions in the semiconductor structure. Incidentally, the fact that there are both current polarities is the reason why such a transistor is denominated as bipolar. On the other hand, MOSFET has certain symmetry in its semiconductor structure, and its operating principle is based on the formation of an electrical charge carrier concentration (a so-called channel) just below the insulator (usually silicon dioxide—SiO_2_) induced by the electric field generated between the gate and the body. The dimension of the channel (in this case n-channel) is proportional to the gate-source voltage (*V_GS_*), and hence the electrical resistance between the drain and source decreases substantially if the bias *V_GS_* exceeds a threshold voltage *V_t_* [8]. There is practically only one current polarity (electrons) through the n-channel and the MOSFET is called unipolar. There are other types of semiconductor junction structures to become a MOSFET.

### 1.2. Principle of Operation of the BJT

**Figure 2:**
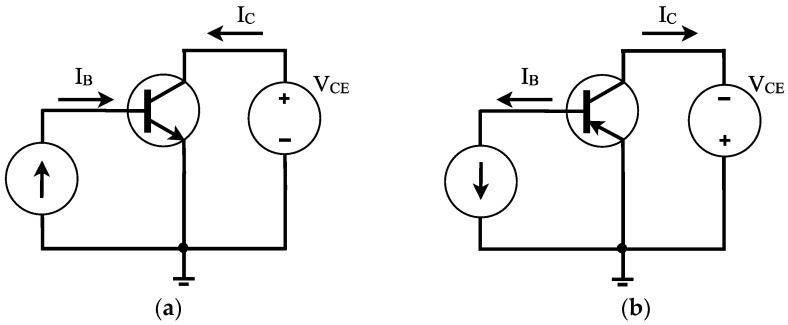
BJT biasing for devices in common-emitter configuration: (**a**) npn; (**b**) pnp.

Figure 2 shows the typical bias for npn and pnp bipolar junction transistors: (1) a current source *I_B_* at the base-emitter junction; (2) a voltage source *V_CE_* between the collector and the emitter. A voltage source with a series resistor can be used to bias the base of the BJT rather than a current source, there is equivalence between them [8,10]; however, in this overview the current source is preferred for reasons to be appreciated later. It can also be seen that the standard symbols represent the flow of the conventional current at the transistor terminals, however it is known that the electron flow is in the opposite direction. The analysis will be based on taking the circuit of Figure 2, which is termed the common-emitter circuit because the emitter terminal is part of both the input signal (*I_B_*) and the output signal (*I_C_*). In this article the common-emitter configuration will often be used.

**Figure 3:**
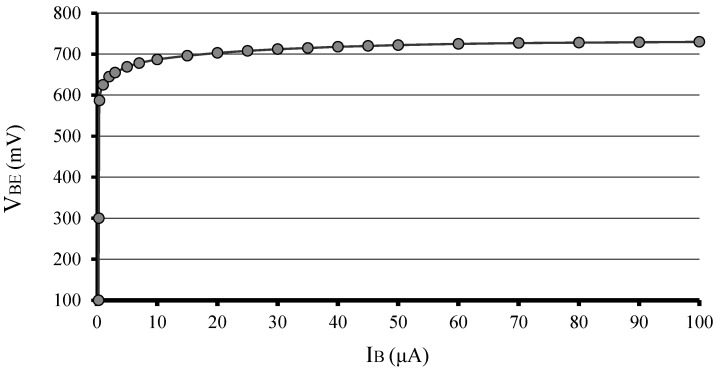
*V_BE_* × *I_B_* curve, with *V_CE_* = 5 V, for a 2N3904 BJT.

To understand how a BJT works, at this point it is interesting to take a simplified approach. First, consider that the BJT is npn type, and the understanding for the pnp it must be to reverse the polarities and exchange electrons for holes. Initially, assume that the base current bias is zero (*I_B_* = 0). This is equivalent to saying that the potential difference and current is zero at the base-emitter pn junction (*J_BE_*), a condition called electronic equilibrium, at the pn junction [5]. This is also equivalent to saying that the base of the transistor is in the virtual ground (a short circuit between the base and the emitter) and the BJT works like a diode. In this case, there is only the reverse bias (*V_CE_*) at the base-collector pn junction (*J_BC_*), so the collector current (*I_C_*) is minimal, in fact practically negligible, because it is an ultra-low leakage current due to the temperature or generation-recombination of charge carriers, for a while. This electrical state (a so-called cutoff) is like an open switch, or it can be said that the electrical resistance between collector and emitter is extremely high (*R_CE_* ≈ GΩ or *R_CE_* ≈ TΩ). Now, in applying a current source bias to the base of the transistor (*I_B_* > 0), there is a direct bias at the *J_BE_*, where a base-emitter voltage *V_BE_* appears, normally referred to as the built-in pn junction potential, *V_pn_* [5]. For a typical silicon BJT, the value of *V_BE_* can be approximately between 0.3 V to 0.8 V depending on the current intensity (nA to mA) through the *J_BE_*, and in general an average value of 0.7 V can be considered for current values in the order of µA. Actually, in the 1960s and 1970s, the radios and TVs that operated with transistors had electrical currents of the order of microamperes in their electronic circuits, and for this reason the engineers used the value of *V_BE_* = 0.7 V as a practical value to design an electronic circuit. Figure 3 shows an experimental curve of a 2N3904 BJT for *V_BE_* × *I_B_*, with *V_CE_* = 5 V, and it can be seen that there is a base current value at which the voltage *V_BE_* ≈ 0.7 V becomes practically constant, like a diode curve if the position of the axes is changed. The curve was obtained with a 2450 Keithley source-meter, in the operating mode called SIMV (current-source and voltage-measurement).

Now let us consider the flow of electrons instead of the conventional current. Then if *I_B_* > 0, it will have the two currents flowing: holes will exit the base to the emitter, and electrons from the emitter enter the base. Naturally, there will be a recombination of electrons with holes, but the base transit time for the electron (*τ_b_*) and the recombination time (*τ*_rec_) are quite different (*τ_b_* << *τ*_rec_). Actually, the bias *I_B_* consists of some components in the innermost structure of the BJT [4]. The first one can be called *I_B_*_1_, which is proportional to *τ_b_*/*τ*_rec_. Therefore, the contribution of *I_B_*_1_ can be very small. Another base current component, *I_B_*_2_, is proportional to $\sqrt{I_{C}}$ and also due to a recombination phenomenon that occurs in the base-emitter depletion region [4]. A third component to be described in this paper is *I_B_*_3_, which depends on device and semiconductor parameters: the ratio emitter to base doping concentration, base thickness, emitter geometry (area and thickness), etc. *I_B3_* is predominant and used to design a BJT [4,5]. According these authors, there are other components in the base current (e.g., tunneling), and it can be difficult to distinguish them, therefore one can assume that:

*I_B_* = *I_B_*_1_ + *I_B_*_2_ + *I_B_*_3_. (1)

**Figure 4:**
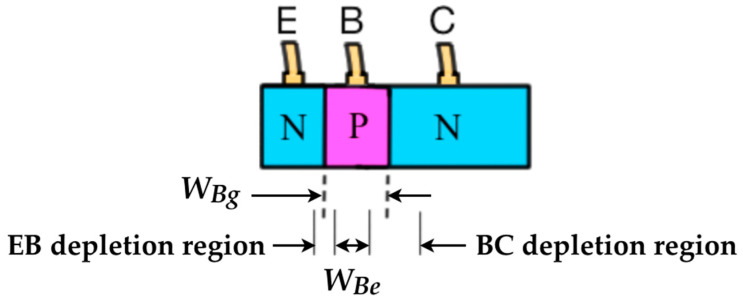
Didactic structure of a BJT to show the difference between *W_Bg_* and *W_Be_*.

Now, if the base current *I_B_* is kept constant, although electrons are the minority charge carriers injected in the base region (for npn BJT), the presence of electrons will maintain a concentration of negative charges there because the hole concentration is lower and there also is electron-hole pairs recombination, and this dynamic of charge carriers in the base modifies the electric field configuration (and consequently the potential configuration) within the innermost transistor structure. Figure 4, which was designed based on the didactic structure of a BJT (Figure 1), is intended to illustrate what is called the neutral base region *W_Be_* (or effective base region) [8], in which the size corresponds to the limits of the depletion regions of the two pn junctions. In this illustration, *W_Bg_* (or simply *W_B_*) is the geometrical base width, which is usually a constant value and normally determined by the transistor fabrication conditions [5].

Generally, *W_Bg_* is very thin and one can didactically say that if an amount of electrons in the p-type base appears there (*I_B_* > 0), then due to the modification of the potential configuration, this means that a lot of electrons can reach the collector region generating *I_C_* >> *I_B_* in an electron multiplication process. That is, the collector current is generated by the injection of minority carriers within the base supplied by: (1) direct bias at the *J_BE_*; and (2) *V_CE_*, which is reversed at the *J_BC_* attracting strongly electrons to the collector. Actually, depending on the BJT biasing, this effect can cause the electrical resistance between the collector and the emitter to decrease abruptly, which can reach extremely low values (*R_CE_* ≈ mΩ or *R_CE_* ≈ Ω), and so the electrons are emitted from the emitter and collected by the collector directly, since the electrical potential of the collector (*V_CE_*) is high enough to accelerate the electrons towards the collector [3]. In fact, inventing a device to switch from the electrical state off to on (and vice versa) was the objective of the research which led to the development of the BJT in 1947 [11]. Incidentally, the term transistor itself derives from this great variation of the electrical resistance (transfer resistor) between collector and emitter. In this way, for common-emitter configuration, the collector current (*I_C_*) is then controlled by the base current bias (*I_B_*) and the most important characteristics in a BJT is the ratio of the collector current and the base current, *I_C_/I_B_*, which is the BJT amplification factor, or more commonly the so-called the transistor gain, *β = I_C_/I_B_*. In electronic engineering, *β* is also called DC current gain *h_FE_*, which is called a hybrid parameter, while the subscript FE stands for forward-emitter in the common-emitter configuration. Italic lowercase subscript is used when the hybrid parameter refers to time-varying signals (*h_fe_* for AC current gain).

**Figure 5:**
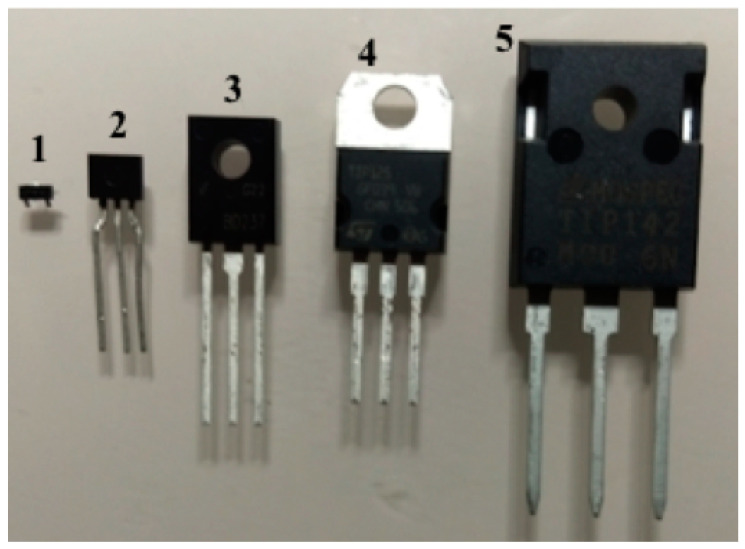
Some types of BJT [and its package]: (**1**) BC846 signal transistor [SMD SOT-23]; (**2**) 2N3904 signal transistor [TO-92]; (**3**) BD237 medium power transistor [TO-126]; (**4**) TIP125 power transistor [TO-220]; (**5**) TIP142 high power transistor [TO-247].

Currently, BJTs are manufactured with DC gains between *h_FE_* ≈ 30 and *h_FE_* ≈ 300; it depends on the type of circuit for which the device will be used: high current (power transistor) or small signal (signal transistor), respectively (Figure 5). A high power transistor is used in industrial applications or in power system circuits (e.g., high power amplifiers), whereas a signal transistor is used in portable equipment (e.g., cell phones).

**Figure 6:**
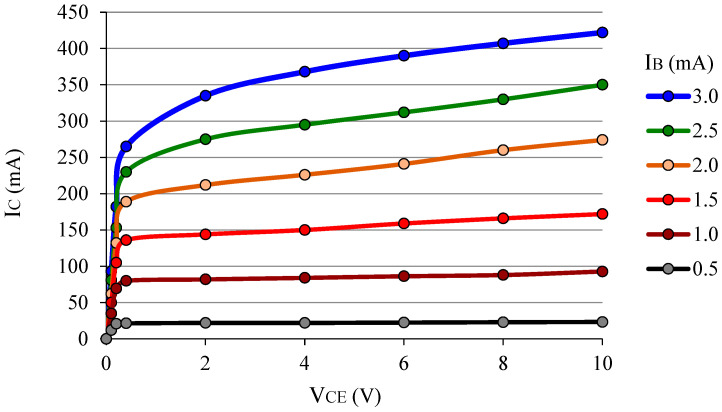
A family of *I_C_* × *V_CE_* curves of a BD237 BJT with *I_B_* as parameter.

In the BJT biasing, the *I_B_* bias will produce *I_C_*, which, associated with *V_CE_*, is called the operating point of a BJT [10]; it is written as *I_C_* @*V_CE_*. Figure 6 shows a family of *I_C_* × *V_CE_* curves of a typical npn BJT (BD237). Note that *I_B_* works as a parameter and there is a *V_CE_* value (≈1 V on the graph) for which *I_C_* increases gradually for a certain range of *V_CE_*. It is important to know that the maximum voltage value to bias the BJT, *V_CE_*_(max)_, known as the breakdown voltage, *V_CEO_*, is generally greater than 30 V and for each type of BJT can be found in the transistor datasheet. This means that if a *V_CE_* greater than *V_CEO_* is applied to the device it can be completely destroyed. There is also a maximum *I_B_* bias which limits the operation of the BJT, but it depends on whether it is a signal or power transistor.

### 1.3. Main Modes of Operation of the BJT

**Figure 7:**
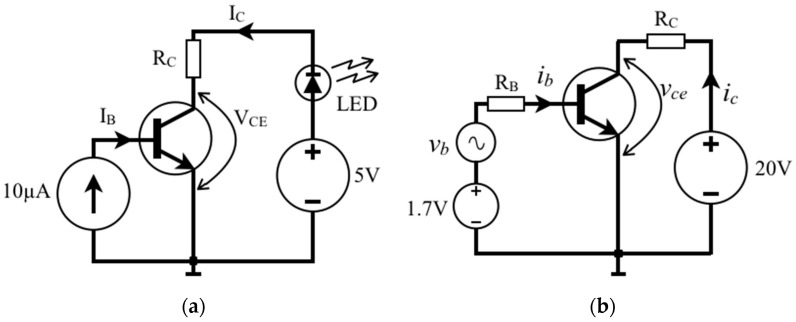
(**a**) Biasing the BJT to light an LED; (**b**) BJT operation to amplify an AC signal, *v_b_*(*t*).

The BJT device can operate in different modes, and the circuit of Figure 7a aims to clarify this topic considering *β* = 200, *R_C_* = 1 kΩ, and assuming that the voltage of the LED in direct bias is constant, *V_pn_* = 1 V. As seen above, if *I_B_* = 0 the LED of the circuit is turned off because the transistor is in the cutoff state (as an open switch), and consequently *I_C_* ≈ 0. Whereas if *I_B_* = 10 µA, for example, then *I_C_* goes to 2 mA and the LED lights up. Precisely in this case one can assume that the operation point of this BJT is *I_C_* = 2 mA@*V_CE_* = 2.0 V, since *V_pn_*_(LED)_ = 1 V and *V_Rc_* = 2 V, and it naturally amplifies the base current so one can say the bipolar transistor is in active mode. If one wants to calculate the power dissipated by the BJT: *P_T_* ≈ *V_CE_*·*I_C_* = 4 mW. Now, increasing *I_B_* just to 25 µA, then *I_C_* tends to go to 5 mA; however, what is obtained as a result of the measurement is *I_C_* ≈ 3.86 mA@*V_CE_* ≈ 0.14 V. In this case, we must note that *I_C_* < *β*·*I_B_*, and it is said that the transistor is saturated, with *V_CE_*_(sat)_ = 0.14 V, which produces *V_Rc_* = 3.86 V. For the BJT, such an electrical state (so-called saturation) is like a closed switch in which the LED is turned on with the maximum brightness possible for this circuit because the *V_CE_* of saturation is reached, and consequently *I_C_* also reaches its maximum value. When a BJT acts exiting from the cutoff to the saturation (or vice versa), it is said that the transistor is operating as a switch: the LED, which was off, turns on (or vice versa). Normally, the transistor operates in active mode when neither is cutoff or saturated, and the equation *I_C_* = *β*·*I_B_* is then true. Figure 6 shows that the transistor is saturated for practically *V_CE_* < 0.5 V [10]. In fact, an electronic engineer designs a sound amplifier circuit so that the BJT is in active mode, so the operating point is kept away (1) from saturation voltage (*V_CE_* > *V_CE_*_(sat)_); and (2) from the cutoff region so that it avoids the harmonic distortion in the amplified sound, for example.

Figure 7b shows another example where there is a time-varying voltage signal source (*v_b_*) in series with the DC bias *V_B_* = 1.7 V, *R_B_* = 100 kΩ, and chose *R_C_* = 5 kΩ. Assuming *β* = 200, *v_b_*(*t*) = 0.5∙sin𝜔*t* (V), and taking *V_BE_* ≈ 0.7 V then the base bias will also vary over time: *i_b_*(*t*) ≈ 10 + 5∙sin𝜔*t* (µA). The output voltage signal at the collector *v_ce_*(*t*) ≈ 10 + 5∙sin𝜔*t* (V) is then obtained. Note that the transistor operating point is 2 mA@10 V, and therefore, as *v_ce_*(*t*) is not less than 5 V (*V_CE_* > *V_CE_*_(sat)_), it is in the active region, resulting in a 20 dB voltage amplifier, however the BJT actually amplifies the current *i_b_*(*t*). On the other hand, to turn on an LED, ideally, the transistor should go to saturation (*V_CE_* ≈ *V_CE_*_(sat)_ or *R_CE_* ≈ mΩ) resulting in very low power dissipation (*P_T_* ≈ *V_CE_*·*I_C_* = *R_CE_*·*I_C_*^2^). Therefore, the mode of operation of the BJT to be chosen depends on the type of circuit: amplification or switching, although in both modes the BJT is a current amplifier.

### 1.4. A Brief Overview on Manufacturing of BJTs

**Figure 8:**
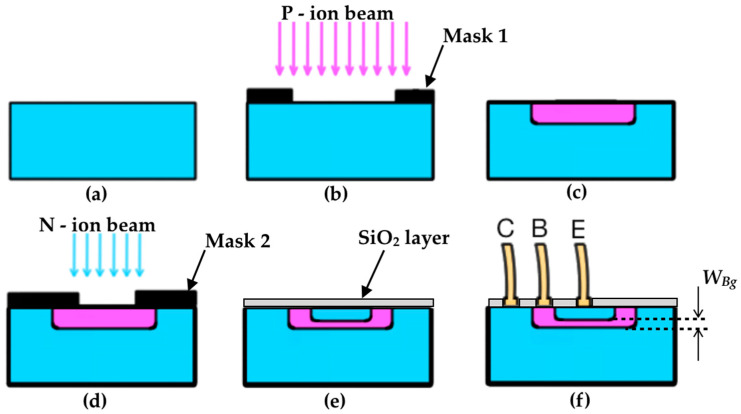
A simplified illustration for the BJT manufacturing process by the double-diffused silicon planar ion implantation: (**a**) n-type Si; (**b**) 1st diffusion; (**c**) p-type diffused on n-type Si; (**d**) 2nd diffusion; (**e**) n-type diffused on p-type Si, and SiO_2_ layer; (**f**) gold wire connections.

The ways of manufacturing transistors are various, such as with the thermal process of diffusion, ion implantation, sputtering, etc. [6,12]. In this overview, the double-diffused silicon planar transistor by ion implantation technique will be summarized [5,13,14]. Actually, the process consists of manufacturing hundreds or thousands of devices practically at the same time in a semiconductor wafer. After manufacturing the wafer containing a large amount of transistors, they are cleaved to become individual transistors. Finally, the transistor structure is metal-wired to the external terminals for use in electronic circuits. Figure 8 displays a practical design for an npn BJT semiconductor structure, and some steps in the manufacturing process are suppressed here for didactic simplification purposes. To facilitate understanding, it is much better to look at how an individual npn transistor is built.

**Figure 9:**
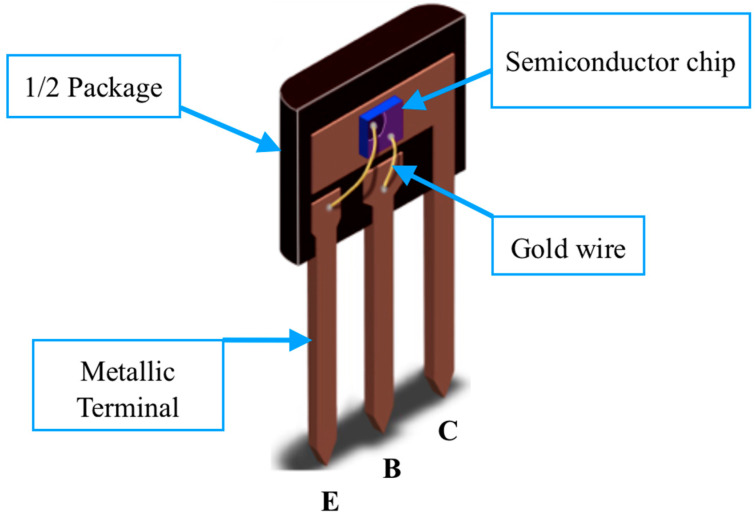
A didactic illustration of the inside of a BJT [16].

Firstly, an n-type silicon chip is exposed to a beam of positive ions to produce the p-type base. The ion diffusion depth can depend on both the energy and the dose of the particle beam applied to the chip. In turn, the ion implantation dose is correlated to the particle intensity and time at which the semiconductor material is exposed to the beam [12]. Also, other aspects inherent to the manufacturing process, as a heat treatment, must be taken into account [15] to provide annealing and minimize semiconductor defects, for example. Secondly, the chip is exposed to another beam of negative ions, and to indicate that the concentration of negative particles in the emitter is larger than in the base, it is usually called an n^+^ doped emitter. Note that in this simplified process the geometrical base thickness, *W_Bg_*, corresponds to the distance between the interfaces of the two pn junctions. There is also a step called the surface passivation process (it could be the first step). Such a step consists of depositing a layer of inert material (often SiO_2_) on top of the chip to protect against changes in the electrical properties of the semiconductor materials. After these initial manufacturing procedure steps of the npn structure, metallization is performed in order to be able to connect the metal wires on the chip. These metal wires, which are often gold, will also be connected on the device’s external metallic terminals, as can be seen in Figure 9.

### 1.5. Variables That Can Change the Output Signal of the BJTs

As reported in the previous sections, there are some parameters that determine the gain of the transistor: base width, emitter geometry, doping ratio at the emitter and base, and other variables [4,5,6]. Some of these parameters of the BJT are usually set at a constant value determined by the manufacturing conditions of the transistor. However, the output signal of a BJT can be altered by some variable, such as: (1) temperature; (2) incidence of light; and (3) its operation point itself.

#### 1.5.1. Temperature

**Figure 10:**
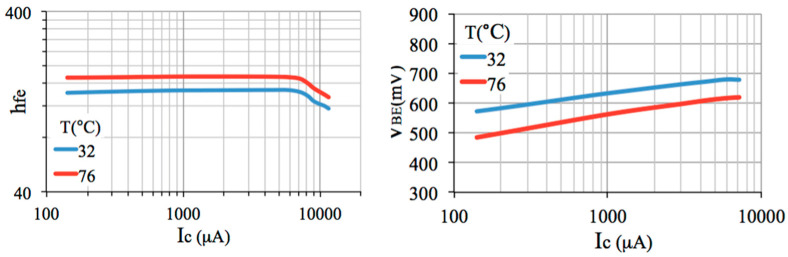
Effect of temperature on a BJT parameters: *h_FE_*, *V_BE_* and *I_C_*.

First of all, according to the electron energy band theory of semiconductor crystal, at room temperature, the effect of lattice vibration produces the generation of electron-hole pairs. So, for intrinsic or extrinsic silicon, when the temperature rises it will result in a combined increasing in the conductivity of electrons and holes. The effect of temperature on a BJT can be found in its datasheet and Figure 10 presents graphs obtained with a 2N3904 BJT for two temperature values (see [17] for more values). Notice that both *h_FE_*, *I_C_* and *V_BE_* are a function of temperature [10]. In addition, it is also observed that each variable depends on the other. As mentioned in the introduction, there are several references containing equations that correlate currents and voltages in the BJT. It is clear that the temperature variation can make the BJT output signal become noisy. In practice, when an engineer designs an electronic circuit with BJT, there is always an electronic component or a small circuit connected to it (as a feedback circuit) providing compensation for the temperature effect. However, if the ambient temperature practically does not vary, as in a typical laboratory, then there is no need for such a compensation circuit.

#### 1.5.2. Incidence of Light

**Figure 11:**
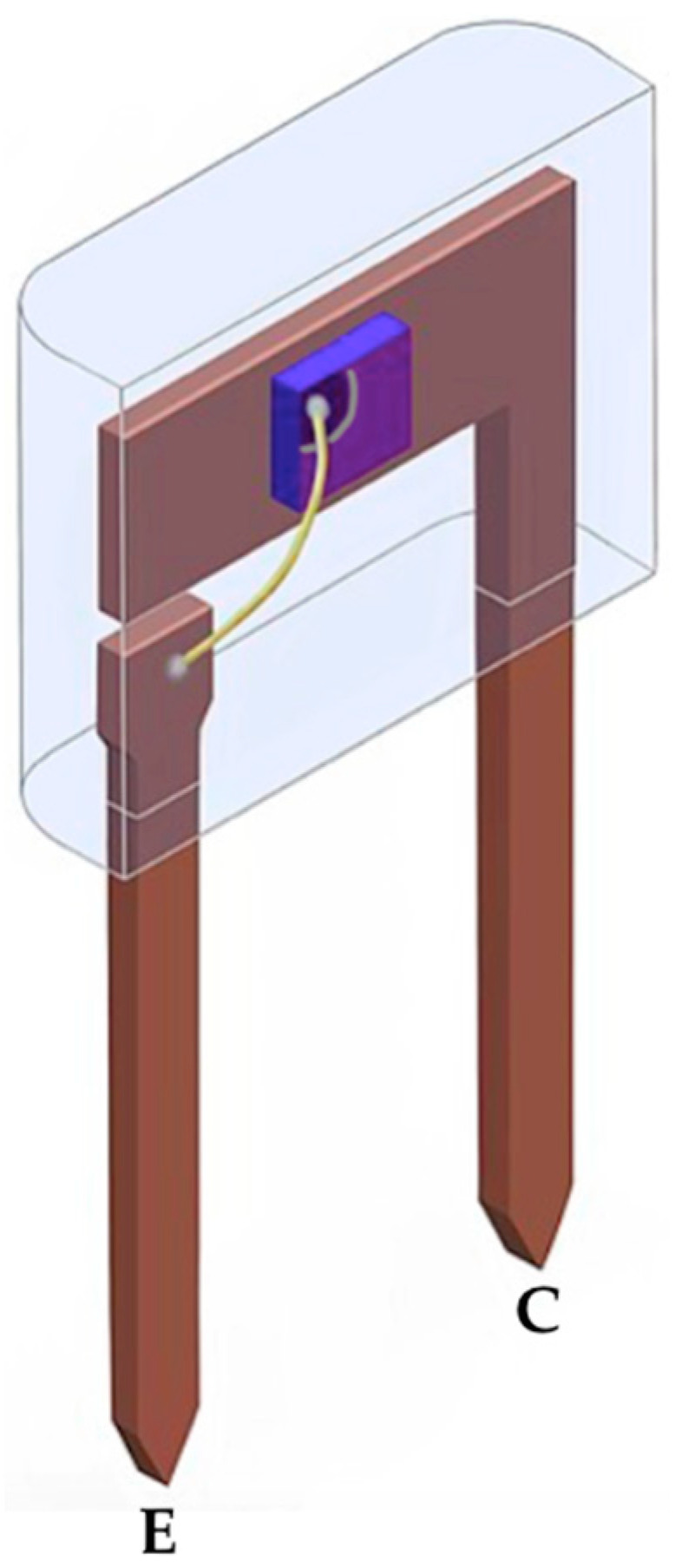
Illustration of a floating base phototransistor [20].

Another variable that could change the output signal of the transistor is the incidence of light. However, in a typical discrete BJT, such an effect does not occur because the package of the device is opaque, and often black. On the other hand, if the encapsulation is transparent, then the device becomes a phototransistor. In this way, the photons of light that reach the chip itself will produce the photoelectric effect at the two pn junctions where the electron-hole pairs at *J_BC_* become the bias *I_B_* which enters into the *J_BE_* and is amplified by gain, *β*. In fact, *J_BC_* works like a photodiode [18,19]. It is worthy of note that, under the same light photon intensity, although the sensitive area of a phototransistor can be 10 times smaller than a typical photodiode, the photocurrent *I_C_* in the phototransistor can be magnified tenfold greater than the photocurrent produced in the photodiode *I_ph_* because of the current gain, *β*. Furthermore, in general the external base terminal of a typical phototransistor does not need to be connected because *J_BC_* works as a photodiode, so there are only the collector and emitter terminals, and the device is known as a floating base phototransistor (Figure 11).

#### 1.5.3. The Operation Point

Another way for the transistor output signal to change undesirably is to alter the operating point itself. Figure 6 shows that the transistor gain gradually increases as a function of *V_CE_* (*V_CE_* > 1 V) for any value of *I_B_*. This is known as the Early effect [10,21], or referred to as base width modulation [5,22]. To understand how this effect can disturb the transistor output signal, one can suppose that there is a constant input signal, *I_B_*, to be amplified by exactly *β* times. However, if the *V_CE_* bias varies then *β* will also vary, and the output current *I_C_* is no longer constant. Then, the *V_CE_* variation will produce a noisy signal at the transistor output. One way to avoid this noisy effect is to apply a well-regulated voltage source to keep *V_CE_* constant. To understand the effect of base width modulation more deeply, one can use Figure 4 and an expression (Equation (2)), which correlates *β* with some parameters of the transistor [6]. The device parameters are: *W_Be_* already defined; *τ_b_* is the minority carrier lifetime in the base; *D_n_* and *D_p_* are the diffusion constant for electrons and holes, respectively; *L_p_* corresponds to the diffusion length for holes in the base, and for npn BJT, *N_A_*/*N_D_* is the ratio base to emitter doping.

(2) $$\frac{1}{\beta }=W_{Be}\cdot \left(\frac{W_{Be}}{2\tau_{b}D_{n}}+\frac{D_{p}}{D_{n}}\frac{1}{L_{p}}\frac{N_{A}}{N_{D}}\right).\;$$

There are two main components whose can alter *β*. The first component corresponds to the recombination phenomenon and the second one is practically correlated to the device parameters. Notice that making *N_D_* >> *N_A_* and minimizing *W_Bg_* (consequently minimizing *W_Be_*) can maximize the gain *β*, and it is actually done in BJT manufacturing processes [6]. Also, if the *V_CE_* bias is increased, then the depletion region of the *J_BC_* is also increased, which actually makes *W_Be_* decrease and consequently increases the gain *β*. If *V_CE_* increases too much, *W_Be_* can become minimal and cause an avalanche of electrons between emitter and collector so that a thermal runaway will occur due to the strong increase in *I_C_* current. That is, the power in the transistor (*P_T_* ≈ *V_CE_*·*I_C_*) may exceed the limit of total power dissipation of the device (*P_D_*) indicated in its datasheet, and then the BJT would be completely destroyed.

### 1.6. Electrical Stress in a BJT

**Figure 12:**
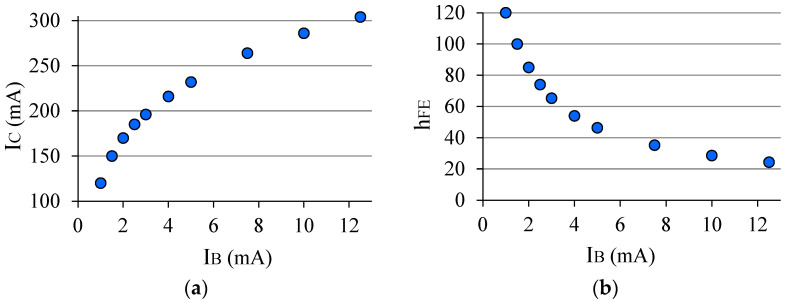
(**a**) *I_C_* during warm-up (5 min); (**b**) *h_FE_* during warm-up.

**Figure 13:**
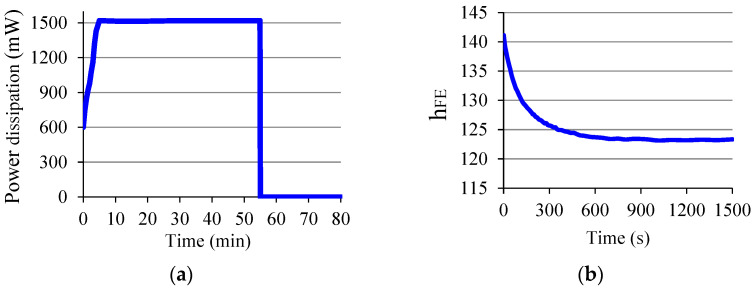
(**a**) Power dissipation in the BJT; (**b**) *h_FE_* immediately after an electrical stress.

The transistor chip may experience mechanical stress during the packaging process, causing changes in the device’s electrical properties [23]. However, what matters most here is the electrical stress, then a brief comment on this subject will be made. There are some conditions that cause electrical stress in a BJT, for example, high base current, reverse bias *V_BE_*, voltage spikes, etc. Toufik [24], in his work, demonstrated the degradation in the BJT gain due to the reverse bias to the *J_BE_*. Here in this paper, a BJT (2N3903) was kept for 50 min with *V_CE_* = 5 V and *I_C_* ≈ 304 mA, dissipating the power of *P_D_* ≈ 1.5 W, which is above the maximum allowed for the device, and for this it was chosen to exceed the maximum collector current, which is *I_C_* = 200 mA [25]. Figure 12a shows how *I_C_* varied in the first five minutes, and Figure 12b shows how *h_FE_* varied during this slow warm-up. Figure 13a shows the curve of the power dissipated by the BJT in the time of electrical stress (50 min). The graph of Figure 13b corresponds to *h_FE_* immediately after the stress period, when the temperature was 115.4 °C in the BJT package. Table 1 brings some initial and final parameters of the BJT. The final gain value was taken 25 min after the electrical stress period in which the device was kept at room temperature. As can be seen in the graph (Figure 13b), after such an electrical stress condition, *h_FE_* increases, unlike what is seen in the case of the electrical stress caused by the reverse bias to the *J_BE_* observed by Toufik [24]. 

Generally, a BJT operating as an X-ray detector works with very low bias currents, and the voltages are below the breakdown limits. Thus, it can be said that BJT under X-ray beams does not experience such an electrical stress. However, the BJT may have changes in its electrical parameters if it is under irradiation by X-ray beams, which will be addressed next.

## 2. Materials and Methods

The source and measurement instruments of electrical quantities in this article are presented in Table 2 and made available by two institutions: (1) The Nuclear Instrumentation Laboratory at CRCN-NE/CNEN; and (2) The Technological Development Laboratory at Scients Company. The Medical Physics and Metrology Laboratories, both also at CRCN-NE provided, respectively, the X-ray beam generators: (1) a Siemens Polymat 30/50 Plus; and (2) a Pantak HF-160. Each experiment was repeated twice to verify reproducibility. Each point on each graph presented hereafter corresponds to an average value of two readings, unless otherwise noted. The relative measurement uncertainties are represented by the size of the marker in each graph and were always less than 0.6% in any experiment performed.

### 2.1. BJT Typical Operation as an X-ray Sensor

A very common device used to measure radiation intensity in diagnostic X-ray beams is the photodiode [26] and also the phototransistor [27]. The BJT has also been used as a detector in diagnostic X-ray and radiotherapy beams [28,29,30]. Before analyzing the BJT operating as an X-ray sensor, a brief description of how a pn junction works under an X-ray photon beam is outlined, taking into account four aspects: (1) X-ray photon energy; (2) device package; (3) scattered radiation; (4) angular dependence.

#### 2.1.1. X-ray Photon Energy

**Figure 14:**
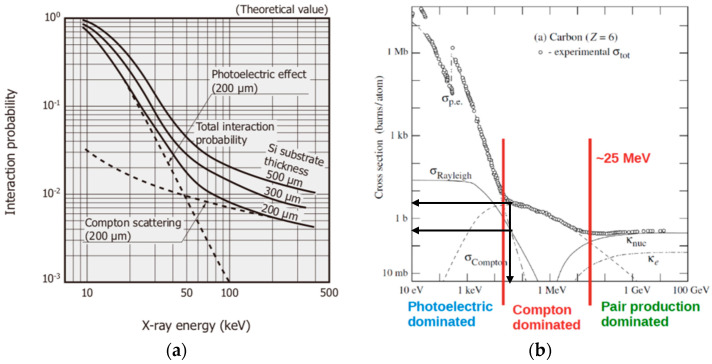
(**a**) Photon interaction probability for silicon [26], courtesy of Hamamatsu Photonics (K.K.); (**b**) Cross section of photons in Carbon [34].

**Figure 15:**
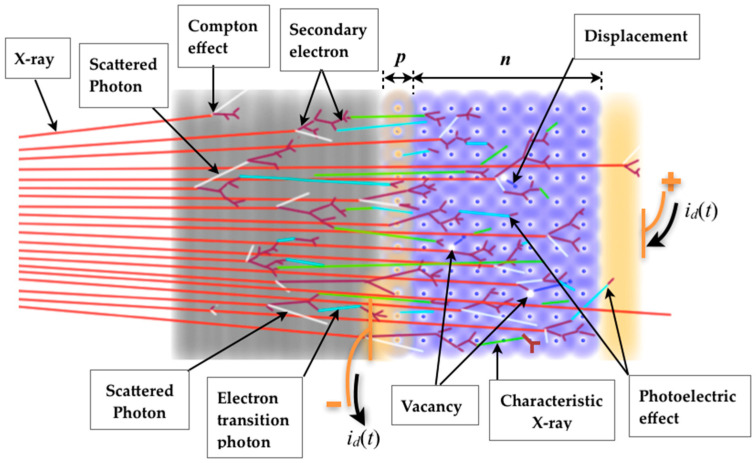
Illustration of a pn junction (reverse biased) and some types of interactions on the chip itself (violet) and its package (grey) where numerous collisions occur, generating the electron rain.

Firstly, consider a device such as a photodiode or a phototransistor which has an encapsulation to filter out ambient light, and only the infrared band (~1.4 eV photons) passes through. Remember that the photodiode has a reverse bias *V_PD_*, and in the case of the phototransistor it also has a reverse bias, *V_CE_*. When the infrared light reaches the photodiode pn junction (*J_BC_* in the phototransistor), the photoelectric effect naturally occurs and a photocurrent called *I_ph_* (*I_C_*) is produced on the photodiode (phototransistor). Without infrared light there will only be a noisy leakage current, *i_n_*, due to the effect of temperature and the generation and recombination of electrons and holes. On the other hand, unlike photons of light, X-ray photons generally have energy of at least four or five orders of magnitude greater than photons of light. The X-ray photon energy will depend on whether the X-ray generating equipment is for examinations of dental radiography (70 kV), conventional radiography (70–125 kV), computed tomography (70–150 kV), etc.; and it also depends on the manufacturer. This means that in the X-ray energy range used in medical diagnosis, the X-ray tube potential can vary between 70 kV and 150 kV (for typical modern medical diagnostic equipment), and it corresponds to X-ray effective energy approximately between 50 keV and 100 keV (see Table 1 in [31]). Actually, the effective energy depends on the X-ray tube potential and the radiation filtration [31,32,33]. In this case, for the photon energy range from 50 keV to 100 keV, the Compton effect predominates, as can be seen in the graphs of Figure 14a,b for the silicon [26] and carbon [34], respectively, the main elements of which the chip itself and its package is made. Figure 14a shows that the probability of the photoelectric effect occurring in 300µm of silicon is less than 3% for energies above 50 keV. Figure 14b is very representative for estimating the probability of photon interactions in materials with a low effective atomic number. For example, above 50 keV the photoelectric effect is at least 10 times smaller than Compton scattering for carbon (see black arrows in graph—Figure 14b). The interactions that occur are with the device as a whole (package + pn junction) generating secondary electrons (so-called electron rain) resulting from both the Compton and photoelectric effect, and multiple electron-electron collisions, as can be seen in Figure 15.

In fact, the Compton scattering is predominant for the energy range used here, however both scattered photons (which have lower energy) by the Compton effect and characteristic X-rays can contribute to the photoelectric effect as well. Such electron rain from the device package and chip itself produce pairs of electron-holes in the semiconductor device, and consequently a flow of charge carriers appears due to the electric field at the pn junction: *i_d_*(*t*), the device current [35].

For photons of light, the currents produced in the photodiode and the phototransistor are called *I_ph_* and *I_C_*, respectively. Because of that, henceforth it is better to differentiate *I_ph_* (*I_C_*) from what it will be called *I_X_* (*I_CX_*), which is the current produced due to X-rays in the photodiode (phototransistor or BJT). It will also be called the bias current *I_BX_* as the virtual base current that arises due to the effect of BJT irradiation. Therefore, hereafter, it will be assumed that the correlation between *I_CX_* and *I_BX_* is:

*I_CX_* = *β*·*I_BX_* ⇒ *I_BX_* = *I_CX_*/*β*. (3)

The device current, *i_d_*, is proportional to both electrical current (*I_XRT_*) and potential (kV) in the X-ray tube. It is known that *I_XRT_* (mA) imposes the photonic intensity of the X-ray beam [32], and the X-ray photon energy spectrum (and consequently both mean and effective photon energy) is determined by the potential (kV). Also, other parameters that determine the X-ray photon energy spectrum are both type of material and thickness of the radiation filtration [32,33].

**Figure 16:**
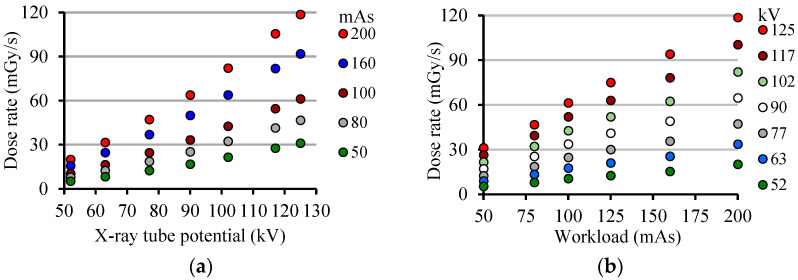
Dose rate $\dot{D}$ at 32 cm from the X-ray focus as a function of: (**a**) kV; (**b**) mAs.

In order to compare some data from semiconductor electronic devices with a reference detector (10X5–6 Radcal ion chamber), the graphs in Figure 16 show the dose rate as a function of the X-ray tube potential (kV) and the workload (mAs) applied to a typical clinical equipment, a Polymat 30/50 Plus by Siemens, which has an inherent radiation filtration of 1 mm (Al). This comparison is easy to make because it is known that *i_d_* is proportional to the dose rate, $\dot{D}$. The dose is then calculated from the integral calculus of the device current, *i_d_*. In Figure 16a, note that $\dot{D}$ is almost linear as a function of the X-ray tube potential between 52 kV and 125 kV. Observe also that $\dot{D}$ is practically linear as a function of workload (Figure 16b). Notice that by selecting the time and the workload on the X-ray equipment panel the value of *I_XRT_* is determined. For example, if 1600 ms time and 80 mAs workload are chosen, then *I_XRT_* = 50 mA. For a time of 1s the workload axis becomes numerically *I_XRT_*.

**Figure 17:**
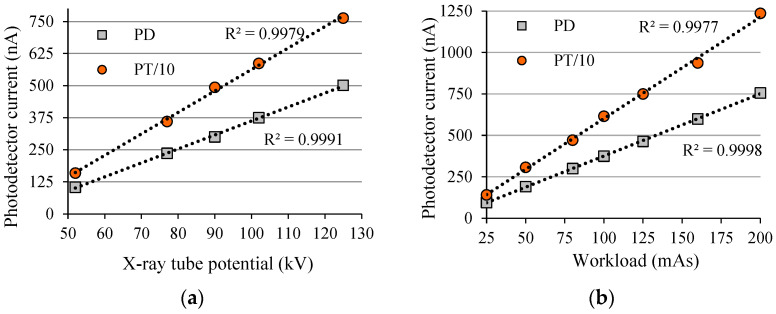
*i_d_* of a photodiode (PD) and a phototransistor (PT), irradiated simultaneously positioned together (at 32 cm), as a function of: (**a**) X-ray tube potential at 100 mAs; (**b**) Workload at 102 kV.

Figure 17 shows how the potential (kV) and current (*I_XRT_*) of an X-ray tube (Siemens, Polymat 30/50 Plus clinical equipment) can alter the value of *i_d_* in two semiconductor electronic devices. In this example, a photodiode (VPT100H) and a phototransistor (TEKT5400S) were placed very close to each other, both positioned at 32 cm from the X-ray focus, and irradiated at the same time. The parameters of the X-ray beam are: 102 kV; 100 mAs. The current of the phototransistor is more than 10 times higher than the photodiode, and because of that the phototransistor current is shown divided by 10 (PT/10). Measurements were taken with an EFF1705 electronic system, Scients.

**Figure 18:**
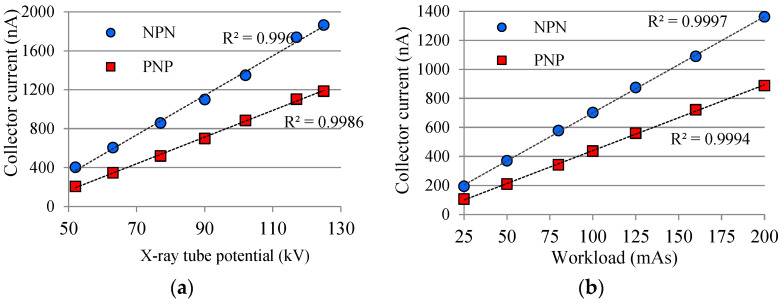
*I_CX_* for npn and pnp BJTs, irradiated simultaneously at the same position (32 cm), as a function of: (**a**) X-ray tube potential (kV@200 mAs); (**b**) Workload (mAs@102 kV).

If the data of Figure 17 are compared with the data of Figure 16, it can be seen that the basic difference is the sensitivity of each photodetector (PD and PT) used for measuring the signal produced by the interaction of radiation on the device. Similar data were previously presented by Valença [36], however the X-ray generator equipment was not one for use in a hospital. Actually, Valença’s experiment was done with metrological equipment, a Pantak HF-320, which has a constant electrical current *I_XRT_* and also an electrical potential (kV) with a very low ripple, i.e., very high precision X-ray generator equipment. Figure 18 shows graphs of device current *i_d_* (in this case *I_CX_*), as a function of two X-ray tube parameters (kV; mAs) for a complementary pair of BJTs: (1) ZTX851 (npn) with *β* ≈ 178; (2) ZTX948 (pnp) with *β* ≈ 113. Such devices are referred to as big chip BJT [37,38]. It can be noted that, like the ion chamber and photodetectors, the linearity of the collector current *I_CX_* is a function of the two X-ray tube parameters (kV; mAs). Actually, the linearity of the response of an electronic sensor is no longer important (for at least 40 years) after the invention of the microprocessor, which can be programmed for measuring any function (exponential, polynomial, etc.).

#### 2.1.2. Device Package

**Figure 19:**
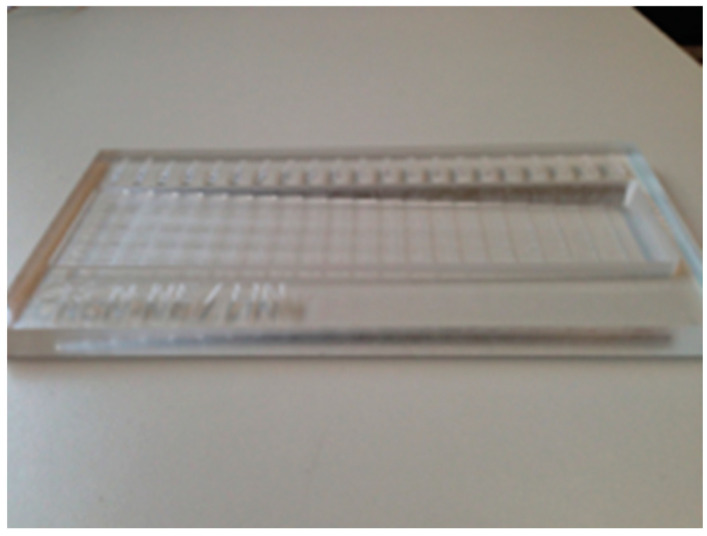
A 0.5 mm step ladder-shaped piece made of PMMA to simulate thicker packages.

**Figure 20:**
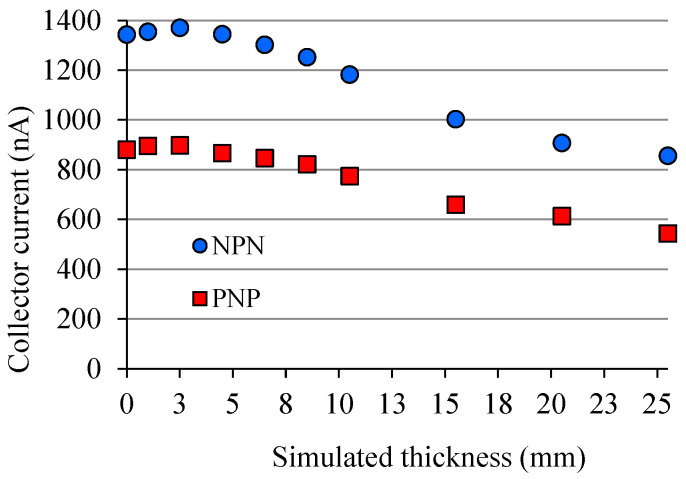
Build-up cap effect for two BJTs, irradiated simultaneously (102 kV@125 mA) at the same position (32 cm), each one has different gains: *β*_1_ ≈ 178 (npn); *β*_2_ ≈ 113 (pnp).

There is influence of the device package in the current signal produced when the device is under diagnostic X-rays. This effect is due to the radiation scattered in front of the semiconductor chip (Figure 15). It is known as the build-up cap effect [39], in which the dose rate increases with the package thickness, *t_p_*. There is a thickness called *t_max_* in which the dose rate reaches its maximum, ${\dot{D}}_{max}$, and if the thickness is greater than *t_max_* the *i_d_* signal decreases. Alves [28] points out in her research that a BC846 signal transistor, which has SMD (Surface-Mount Device) package, presents *t_max_* of around 5 mm. To perform her experiment, the thickness was varied using a 0.5 mm step ladder-shaped piece made of PMMA (Figure 19) to gradually simulate a thicker package. The material (PMMA) was chosen because it has an approximate effective atomic number of the material that the BJT package is normally made of. To perform such an experiment, the piece was positioned tightly against the transistor. The same piece from Alves’ experiment [28] and another 3 pieces of PMMA, 5 mm tick, were used to illustrate graphically how the thickness of the package can influence the *I_CX_* signal produced by a complementary pair of BJT, and Figure 20 shows data from these BJTs simultaneously irradiated. Each BJT has different gains: *β*_1_ ≈ 178 (npn); *β*_2_ ≈ 113 (pnp).

Looking at the graph in Figure 20, it can be seen that the curve shape of each BJT is very similar regardless of the gain and polarity of each one. That is, the *I_CX_* current increases with the increase in the thickness of the simulated package and then from *t_p_* ≈ 4 mm onwards *I_CX_* starts to decrease. The difference in the Alves’ result and those in Figure 20 is that the BC846 device used by Alves [28] has a SOT-123 SMD package, which is about 1 mm thinner than the big chip BJT used here, then the difference in result was expected.

#### 2.1.3. Scattered Radiation

**Figure 21:**
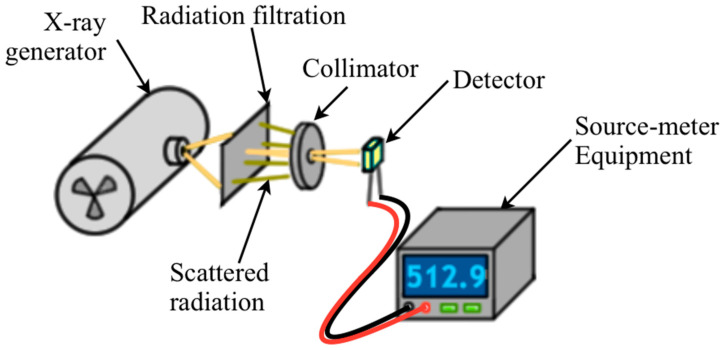
Illustration of experimental setup to analyze the effect of the scattered radiation in a BJT.

It must be evaluated whether scattered radiation far from the device can be significant in the radiation measurement procedure with BJTs. Such an evaluation can be important if the device is used to measure only primary radiation (e.g., non-invasive kV measurement). One way to analyze such an effect is to place a radiation filtration plate at a distance from the device (Figure 21). By placing a collimator between the plate and the device under radiation, it is possible to verify how much the scattered photons on the plate are measured or not by a BJT. Such an experiment can be performed by varying the area of the collimator for measuring how *I_CX_* varies as a function of collimation (Table 3).

Table 3 indicates that *I_CX_* practically varies about ±1.5% for the areas of collimators between 100 cm^2^ and 16 cm^2^. Comparing the readings for 100 cm^2^ and 1 cm^2^ it can be seen that the contribution of scattered radiation is around 7%. The last column of Table 3 shows that the area of the radiation field is practically the area of the device package. For the collimation which exactly covers the transistor package (~0.2 cm^2^), the contribution of scattered radiation is about 11% in relation to 100 cm^2^. In the previous approach on the build-up cap effect, it was observed that the scattered radiation near the semiconductor chip contributes to the increase in *I_CX_*. However, if the BJT is used to measure the dose at a specific point, as in the case of a dosimeter, it must measure both direct and scattered contributions delivered to a patient undergoing examination. That is, as the BJT is a very small electronic component, it becomes advantageous to perform high accuracy dosimetry studies in phantoms to then obtain data without the need for human beings, for example.

#### 2.1.4. Angular Dependence

Angular dependence corresponds to an analysis of how the detector signal varies as a function of the angle of incidence of the X-ray beam on the device face. To assess the angular effect, it is enough to vary the angle of the normal vector to the chip plane in degrees, for example, as Alves [28] also did. Table 4 presents an angular dependence for a BJT and it is similar to what Alves [28] found. Note that in rotating the device clockwise the signal is slightly less than when rotating it counterclockwise. Actually, in addition to the fact that the chip area decreases in relation to the direction of the incident radiation beam, it is due to two effects: (1) the X-ray path is increased when the device is rotated, resulting in the build-up cap effect; and (2) the connecting wire between the emitter and its own external metallic terminal (see Figure 9) causes a slight attenuation of the radiation beam that reaches the *J_BC_* if the device is rotated clockwise.

All data presented in the approaches above can be found in the literature, although some of them were obtained especially for this article. The main devices used in this article consist of complementary pairs of BJTs with several different gains: ZTX851 (npn) and ZTX948 (pnp), which have an E-Line type package (TO-92 compatible). Another complementary pair of BJTs, with package TO-220, were also used only for comparison purposes: TIP41 (npn) and TIP42 (pnp). Before presenting the main results, it will be shown how these BJTs were biased and how to obtain the collector current *I_CX_*.

### 2.2. Method for Measuring I_CX_ in a BJT

**Figure 22:**
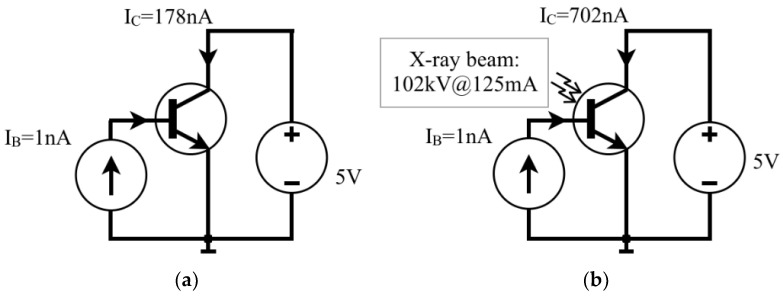
(**a**) Biasing a BJT with *β* =178 before irradiation. (**b**) *I_C_* during irradiation.

First of all, before irradiating the transistor, it is electrically biased to help us understand how the BJT works as an X-ray sensor. Therefore, an experiment is done by biasing the transistor with an ultra-low *I_B_* value (e.g., 1 nA) so that there is an initial collector current *I_C_* ≈ 178 nA, for a ZTX851 BJT with *β* ≈ 178 as shown in Figure 22a. Second, Figure 22b illustrates the biased BJT under an X-ray beam and this results in *I_C_* = 702 nA. The calculation of *I_CX_* must be done by subtracting the component of the initial collector current *I_C_* = 178 nA, then *I_CX_* = 524 nA, for the example. From the value of *I_CX_*, one can calculate the value of *I_BX_* ≈ 2.9 nA, which would correspond to a base current component only due to X-rays. Thus, it is assumed that *I_BX_* was supposedly amplified by *β*, as previously shown in Equation (3). In this approach, the parameters of the X-ray tube were 102 kV; 200 mAs; and 1600 ms, resulting in an X-ray tube current of 125 mA, and the device was positioned 32 cm from the X-ray source.

## 3. Results

Taking into account the approaches above, it will be explained how the BJT behaves when under X-ray beams used in the medical diagnostic energy range. It is assumed in this explanation that the BJT is the planar type as described in the BJT fabrication overview.

### 3.1. Influence of I_B_ for a BJT Operating as an X-ray Sensor

**Figure 23:**
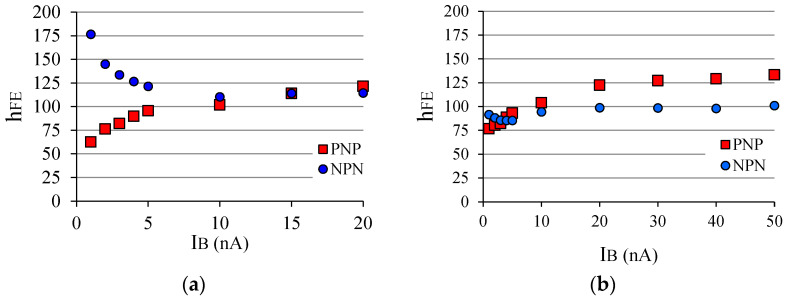
(**a**) Measured gain behavior of a complementary pair of BJTs with *β*_1_ = *β*_2_ @ 15 nA; (**b**) Measured gain behavior of a complementary pair of BJTs with *β*_1_ ≈ *β*_2_ @ 4 nA.

In the previous analysis, a bias of *I_B_* = 1 nA was used. However, if one wants to know how much *I_B_* influences the sensitivity of BJT to X-ray beams, it is necessary to vary *I_B_*. First, the BJT gain behavior without any irradiation is presented in Figure 23 for two complementary pairs of BJTs. For very low *I_B_* values (between 1 nA and 5 nA), the gains of the two devices vary significantly. However, after 10 nA they vary much less than for low *I_B_*, for both types of transistors. Second, another experiment was made to verify how complementary BJTs behave when under irradiation. To perform such an experiment, a pair of BJTs (*β*_1_ ≈ 101, npn; *β*_2_ ≈ 101, pnp; for *I_B_* = 10 nA) never before irradiated were selected. Table 5 presents the results, and *h_FE_* corresponds to the gain value for *I_B_* without X-ray incidence on the BJT. *I_CX_* was measured and *I_BX_* calculated from Equation (3). Note that *I_CX_* is not the same for every value of *I_B_*, and there does not appear to be a monotonic correlation, although *I_CX_* increases with *I_B_*.

By analyzing in a general way the results of Figure 23 and Table 5, one can see that: (1) there is a certain influence of *I_B_* on the transistor gain (with or without radiation); (2) *I_BX_* is higher in the npn transistor than in the pnp transistor, i.e., npn BJT seems to be more sensitive to X-ray beams normally used in medical diagnosis; (3) *I_BX_* of the pnp BJT reaches the highest value for *I_B_* = 10 nA, whereas for the npn BJT it occurs at *I_B_* = 40 nA. In fact, it was seen earlier in Figure 10 that *I_C_* (and hence *I_B_*) varies with the transistor gain and vice versa, so this is nothing new. However, there seems to be a doubt about *I_BX_* because Table 5 provides estimated values for the virtual bias, *I_BX_*, since the current *I_BX_* itself influences the gain and vice versa. Actually, there is a way to measure *I_BX_*. It is known that X-ray photons reach the chip itself generating electron-hole pairs there, and this contributes to a fraction of *I_CX_*. Remember that the device is irradiated as a whole. Then, knowing that the Compton effect is predominant in the package, part of the electron rain from the package reaches both the *J_BE_* and the *J_BC_*. Looking at Figure 8, one can see the *J_BE_* is just above *J_BC_* and it is the first region on the chip itself that receives the electron rain from the transistor package. Another fraction of radiation (photons and secondary electrons) reaches the back of the device (usually a metal substrate) and probably does not contribute significantly to *I_CX_*. From this point of view, *I_CX_* is formed by a complex combination of Compton and photoelectric effects, and interactions from scattered photons and secondary electrons from both package and chip itself (see Figure 15). In this paper, what matters is the result of that combination of all components that will act as the virtual base bias, *I_BX_*, which is supposedly amplified by *β*, and it can be assumed that:

*I_EX_* = *I_CX_* + *I_BX_* = (*β* + 1)·*I_BX_* (4)

*I_BX_* = *I_BX_*(*J_BC_*) + *I_BX_*(*J_BE_*). (5)

Equation (4) is known and derives from charge conservation (Kirchhoff’s law), whereas Equation (5) is to propose separating *I_BX_* into two components: (1) in the *J_BE_*; (2) in the *J_BC_*.

**Figure 24:**
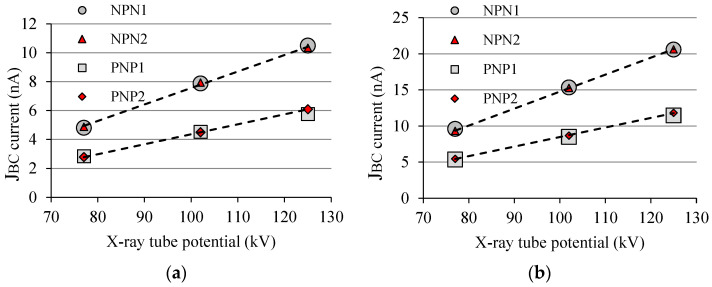
*I_BX_*(*J_BC_*) measurement (short-circuiting in the *J_BE_*) as a function of X-ray tube parameters for two complementary pairs of BJTs (ZTX851 & ZTX948): (**a**) 100 mAs; (**b**) 200 mAs.

Then, making a short-circuit in the *J_BE_*, one can estimate the contribution only due to *J_BC_* and then know directly the value of *I_BX_*(*J_BC_*) from Equation (5), since in this case *I_BX_*(*J_BE_*) = 0. When the transistor is irradiated without short-circuiting in the *J_BE_*, it is then possible to estimate *I_BX_*(*J_BE_*) from Equation (5) with the previous values of *I_BX_*(*J_BC_*) when there was a short-circuiting in the *J_BE_*. For comparison purposes, two complementary pairs of BJTs were irradiated simultaneously (each pair at a time) in order to compare whether *I_BX_*(*J_BE_*) or *I_BX_*(*J_BC_*) are significantly different from each other. For *I_B_* ≈ 10 nA, the first pair has gains of 91 (NPN1) and 99 (PNP1); and the other pair has gains of 83 (NPN2) and 84 (PNP2). Figure 24 shows graphically the results of *I_BX_*(*J_BC_*) as a function of the X-ray tube parameters (kV and mAs). Notice that the response of each transistor does not correlate with the gain since the *J_BE_* is short-circuited.

**Figure 25:**
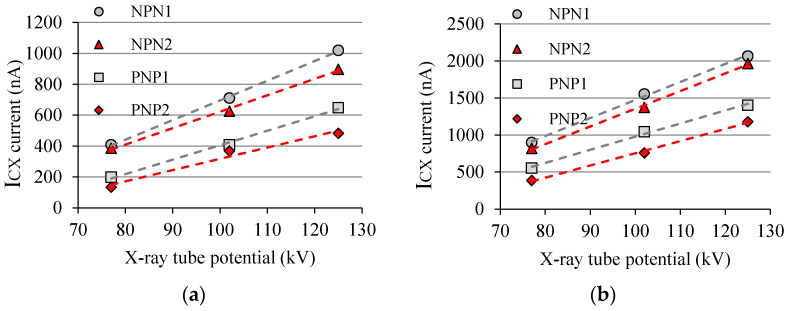
*I_CX_* as a function of X-ray tube parameters without short-circuit in the *J_BE_*: (**a**) 100 mAs; (**b**) 200 mAs.

The *J_BC_* component varies linearly with both parameters (kV and mAs) similarly to BJT under radiation with *I_B_* ≠ 0 (see Figure 18). Furthermore, the sensitivity is practically the same for each type of BJT, i.e., although the gains are different, the trend line is practically the same. Figure 25 brings the response to the X-ray beam from each device in Figure 24 without a short circuit in the *J_BE_*, and it can be seen that each device has its sensitivity according to the gain and its own characteristics. Transistor package, current gain, and chip doping are typical examples of BJT parameters that can make the electrical response of a device to X-rays different from a similar one.

Table 6 and Table 7 present some values of the *J_BC_* component and the respective values of *J_BE_*, calculated from Equation (5), for another complementary pair of BJTs, both with *β* ≈ 100. Note that for any X-ray tube parameters the behavior trend is similar. Moreover, observe that for both npn and pnp transistors the *J_BE_* component is smaller than the *J_BC_* component, mainly for the npn transistor, which is about 10% on average. This is correlated with the fact that *J_BE_* is a thin film on the chip surface, whereas *J_BC_* consists of most of the transistor volume. Even knowing that for each BJT operating as an X-ray sensor has its own characteristics and the fact that *β* can vary with *I_B_*, consolidating the results from Figure 24 and Table 6 and Table 7 leads to the conclusion that *I_BX_* actually works as a base current bias in parallel to *I_B_*. This is why it was decided to use *I_B_* bias as a current source rather than a voltage source in Section 1.2.

The statement above becomes even more evident if another experiment is performed, by varying the X-ray tube potential (kV) to inject into the base a value of *I_B_* equal to −*I_BX_* for each X-ray tube potential. Performing this technique, it was verified that each *I_BX_* value is then canceled resulting in each value of *I_CX_* ≈ 0 for each kV value. To guarantee nullifying the effect of *I_BX_*, it was necessary to inject values of *I_B_* ≈ −1.05∙*I_BX_*, i.e., about 5% of the estimated value of *I_BX_* in Table 6 and Table 7. Actually, this technique became a way to determine the peak potential value of the X-ray tube (kVp) for which the patent application was made [40].

### 3.2. Radiation Damage in BJT as an X-ray Sensor

**Figure 26:**
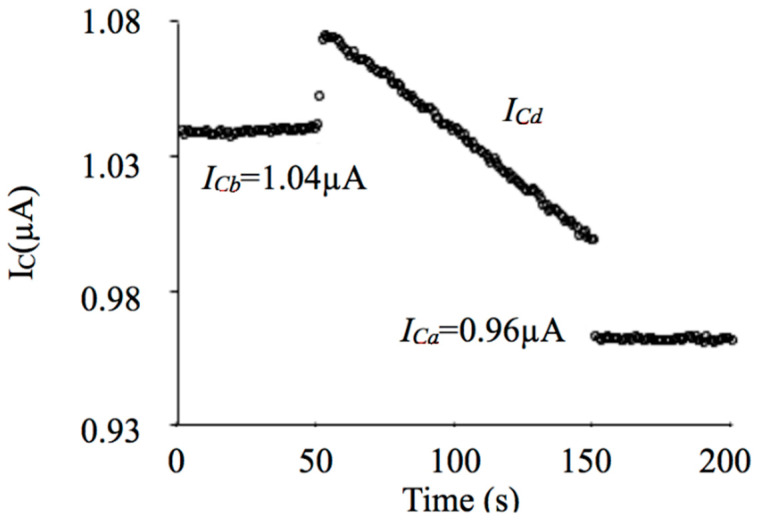
*I_C_* of a 2N3904 BJT before (*I_Cb_*), during (*I_Cd_*) and after (*I_Ca_*) exposure to standard X-rays.

The last topic in this overview concerns the radiation damage in BJT. It is well known that if a BJT is exposed to radiation its electrical characteristics can change, and it depends on the type and energy of the particle, and also on the radiation dose received by the device [41,42]. Basically, following these authors and others [43,44,45], two types of damage in BJT can be considered as the most important: ionization and displacement. The radiation-induced defects can occur in the bulk semiconductor (Si), insulating layer (SiO_2_), or Si-SiO_2_ interface [41,42,43,44,45]. The damage can be unstable and it can also be neutralized under heat treatment, for example [9,41,42,43,44,45]. However, in this paper what matters is the resulting effect of all radiation damage components on the device, which can modify the electrical parameters of the BJT. In this way, it can be seen in Figure 26 [29] that there is variation of the collector current of a signal transistor in real-time: before, during and immediately after exposure to X-rays. The graph (extracted from [29]) was based on measurements made with a 2N3904 BJT, with *β*_0_ ≈ 104, *I_B_* ≈ 10 nA, under continuous X-ray beam, generated by the Pantak HF-320 equipment, with the potential of 100 kV, an X-ray beam spectrum originated by an aluminum radiation filter with a thickness of 2.5 mm, and the dose rate of around 10 mGy/s. Note that the step up in the value of *I_C_* before irradiation is practically equal to the step down after irradiation, about 38 nA, which was previously named as *I_CX_*. Then, one can say that *I_BX_* ≈ 0.37 nA. Furthermore, looking at *I_C_*, it is important to note that the electrical state of the transistor (i.e., *h_FE_*) systematically changes during the irradiation procedure, since *I_B_* and *I_BX_* are theoretically constants.

**Figure 27:**
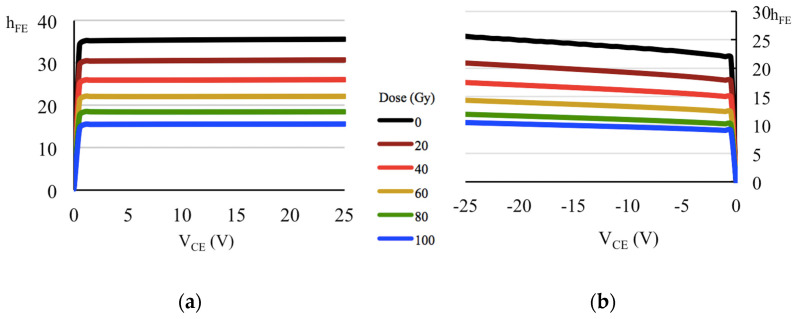
*h_FE_* as a function of *V_CE_* for a pair of BJTs (nominal *β* ≈ 30 [48,49]): (**a**) TIP41; (**b**) TIP42.

**Figure 28:**
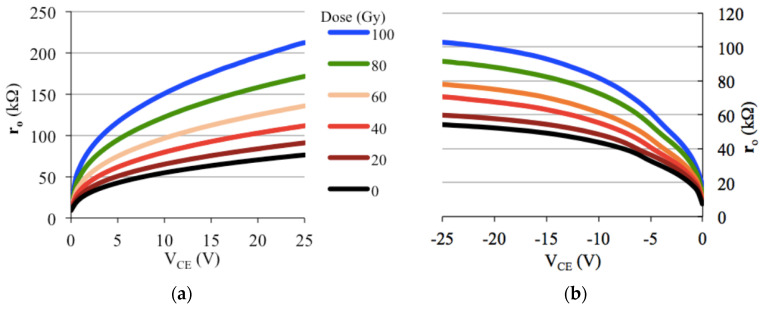
*r_o_* as a function of *V_CE_* for a pair of BJTs (nominal *β* ≈ 30 [48,49]): (**a**) TIP41; (**b**) TIP42.

From the last result, in a practical and didactic way, one can say it is the parameter *h_FE_* of the transistor which systematically decreases with the accumulated dose of X-rays in the energy range used in medical diagnosis, as can be seen in Figure 27. This experiment consisted in reproducing the technique used by Monte [46,47] to measure the gain *h_FE_* and the output resistance, *r_o_*, of a complementary pair of BJTs (TIP 41 and TIP 42), nominal *h_FE_* ≈ 30 [48,49]. The experiment was performed with the Pantak HF-160 X-ray generator (100 kV@10 mA; 1 mm Al radiation filter), which can bombard the device with X-ray photons continuously since it has a coolant for continuous cooling. Figure 28 shows that *r_o_* increases after the accumulated dose up to 100 Gy on the devices. Other authors [42] also report an increase in the collector resistance, which is *r_o_*, assuming the small-signal equivalent circuit [10,46] that is due to radiation-induced defects in the semiconductor region corresponding to the collector. Table 8 summarizes the behavior of the parameters *h_FE_* and *r_o_* (for |*V_CE_*| = 25 V) on the complementary pair of BJTs, parameterized with the accumulated dose of an X-ray beam in the energy range of the diagnostic: the BJT gain decreases whereas the output resistance increases.

**Figure 29:**
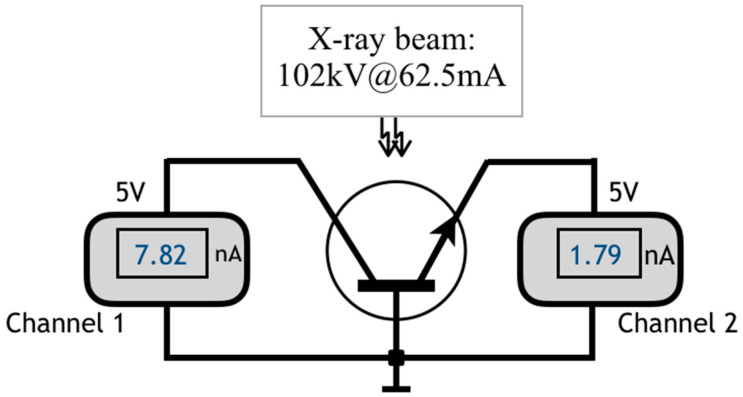
Circuit used for a reverse biased *J_BC_* and *J_BE_* irradiation procedure to analyze how the transistor behaves during 40 exposures with X-ray tube parameters selected to be 102 kV@100 mAs.

Regarding the BJT input resistance, here called *r_in_*, which can be called *R_BE_* or *r*_𝜋_ in the small-signal equivalent circuit [10,46], some authors also report its increase after irradiating BJT under high energy particles (see [42] and references therein). In order to verify how *r_in_* and *r_o_* of a BJT behaves during a sequence of 40 exposures of the device in a typical diagnostic X-ray photon beam of a clinical equipment (Siemens, Polymat 30/50 Plus; with 102 kV@62.5 mA), another experiment was performed with a ZTX851 device never before irradiated, *h_FE_* ≈ 100, of which the experimental setup is presented in Figure 29.

**Figure 30:**
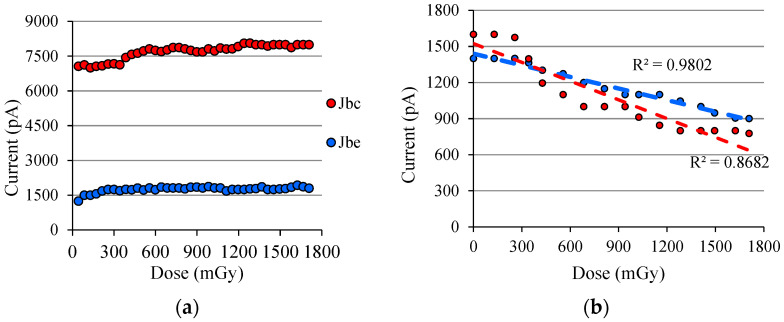
(**a**) Behavior of the *J_BC_* and *J_BE_* currents during exposures (for the circuit in Figure 29); (**b**) Behavior of the *J_BC_* and *J_BE_* currents between intervals in which the X-ray beam is off.

The BJT has been reverse biased to both *J_BC_* and *J_BE_*, each junction on each source- meter instrument was measured simultaneously. The first results are shown in Figure 30a, and it can be seen that both junction currents at the time of exposure to the X-ray beam do not change significantly after the fifth and twelfth exposure for *J_BE_* and *J_BC_*, respectively. Figure 30b shows the results of the BJT junction behavior during the time interval when the X-ray beam is off, which is for approximately nine minutes between every three X-ray shots.

Note that the *J_BC_* reverse current systematically decreases (red trend line), which suggests that the *r_o_* resistance increases. The graph also shows that the reverse current in *J_BE_* decreases (blue trend line), thus suggesting that *r_in_* also increases. Although the trend of *r_in_* and *r_o_* were shown (dashed lines), the determination of these parameters is complex for the following reasons: (1) the collector resistance (and then *r_o_*) is strongly dependent on the device operating point [10], as can be seen in Figure 26; and (2) the emitter resistance is not unique, as it depends on the base current [9].

Some authors formulate the effect of BJT gain degradation as the decrease in collector current with the increase in some component of the BJT base current (Equation (1)), which is commonly called excess base current [50,51,52,53]. Also, Equation (2), which presents some variables or parameters of the device, can be (for a certain type of radiation) the source of variation of the BJT gain. However, for simplicity, such an analysis is not adequate here. Taking into account that *I_B_* is kept constant, it will be assumed that the damages produced by radiation on the BJT, in all parts of the device, lead to a decrease in the gain and consequently in the collector current. In a practical way, one can didactically say that the irradiation of diagnostic X-ray beams in the BJT changes the electrical state of the device, and one way to determine its new electrical state is to measure *h_FE_* (or *I_C_*), for example. Looking from the point of view of the inverse problem, by measuring the new electrical state of the device one can then determine the radiation dose received by the device, which was a suggestion given by Monte [46,47].

### 3.3. Repeatability in Measurements Made with BJT as an X-ray Sensor

At this point, it is important to evaluate the following aspects: (1) What is the repeatability the *I_CX_* signal provides under diagnostic X-rays?; and (2) For typical dose values in some radiograph examinations, after repeated use of the BJT as a sensor, how much does the BJT gain degrade?

**Figure 31:**
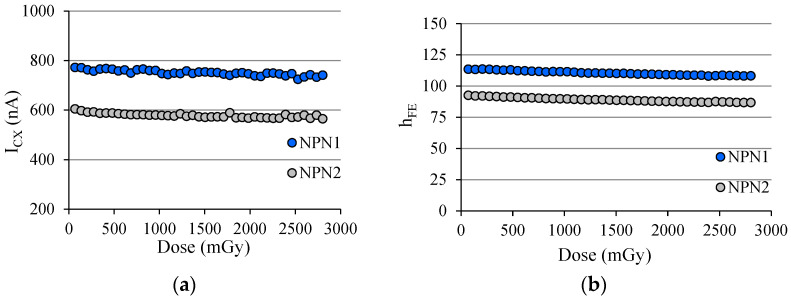
(**a**) *I_CX_* behavior of two npn BJTs during a sequence of 40 exposures to an X-ray beam with the X-ray tube parameters: 102 kV@100 mAs; (**b**) *h_FE_* behavior of the same pair of BJTs.

Regarding the first question, Figure 31a shows the *I_CX_* behavior for two npn BJTs both biased with *I_B_* ≈ 5 nA, which were exposed to an X-ray beam (102 kV@100 mAs), and the dose rate is set to be $\dot{D}\;$= 42.7 mGy/s. Each marker corresponds to the average of three measurements of *I_CX_* during the X-ray shot, which had a duration of 1600 ms. Figure 31b shows the *h_FE_* behavior for the same pair of transistors, and each marker is the average of 180 measurements of *h_FE_* corresponding to the time interval (3 min) in which the X-ray tube is cooling down.

**Figure 32:**
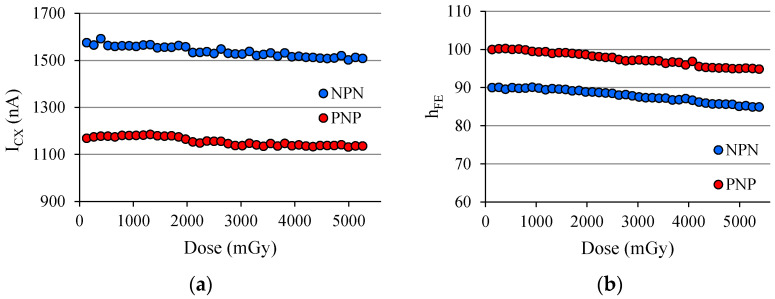
(**a**) *I_CX_* behavior of a complementary pair of BJTs during a sequence of 40 exposures to an X-ray beam with the parameters: 102 kV@200 mAs; (**b**) *h_FE_* behavior of the same pair of BJTs.

For both transistors, regardless of *h_FE_*, the degradation was approximately −5% for the accumulated dose of around 2.8 Gy. Figure 32 was originated from the analysis of a complementary pair of BJTs (ZTX851 & ZTX948) both biased with *I_B_* ≈ 10 nA irradiated simultaneously under an X-ray beam with parameters to be 102 kV@200 mAs, and the dose rate was $\dot{D\;}$ = 82.1 mGy/s. The duration of the X-ray shot was also 1600 ms.

Note that the dose rate received by the devices in Figure 32 is nearly double that of Figure 31, and the *h_FE_* variation was also approximately −5%. Basically, the difference between these two experiments consists of *I_B_*, *D*, and $\dot{D}$; both parameters were doubled.

## 4. Discussion

As the results of percentage gain degradation were similar, it is noted that the lower dose rate experiment produced a more significant effect on the BJT. The dose rate dependence of gain degradation on BJT under radiation was already analyzed [50]. Furthermore, the irradiation of BJTs with and without bias was also recently analyzed [46], and it was observed that there is a difference in the results. These results lead to the conclusion that both *I_B_* and $\dot{D}$ can influence changes in the transistor gain when irradiated.

According to these last two graphs, the answer to the first question is: BJT gain degradation is a function of the dose received by the device and can be measured from the value of *h_FE_* (and hence *I_C_*), which varied about −5% after approximately 5.6 Gy. From these results, as well as Table 8, in which TO-220 package BJTs were analyzed and where *h_FE_* degradation could be estimated about −5% after approximately a 7 Gy accumulated dose, one can say that both types of BJT and their packages play an important role in measuring diagnostic X-ray beams applied to medical diagnosis.

Based on all results presented here, it is important to comment on the limitations of using BJT as a sensor in diagnostic X-ray beams. Therefore, the answer to the second question from the previous subsection can be elaborated on by taking into account the dose delivered to a patient, *D_pat_*, in a typical radiograph examination. At present, *D_pat_* can vary between 0.4 and 40 mGy [54], depending on whether it is a posterior-anterior (PA) projection, lateral (LAT) projection, etc. It also depends on the type of organ: chest (~0.9 mGy), skull (~4 mGy), dental (~6 mGy), lumbar spine (~27 mGy), etc. Knowing that the most frequent radiographic examination is that of the chest, one can assume that the weighted average dose is approximately less than 4 mGy (~3.5 mGy). Although the response of the BJT to the X-ray beam depends on certain conditions such as the bias, the dose rate, the radiation filter (X-ray beam spectrum), etc., for the purpose of just having an example, for a BJT to operate as an X-ray sensor continuously receiving an average dose of 3.5 mGy (each X-ray shot), the error in collector current would reach about −5% (≈7 Gy for TIP41 at *V_CE_* = 25 V) only after about 2000 radiograph examinations. This could mean changing the device after about 10 weeks in a typical radiography clinic, or at least a recalibration process should be made within that time period. If the BJT is used once a week for radiation beam monitoring purposes then it could be used for up to one year before the first recalibration.

Finally, there are two additional comments arising from the effect of radiation on BJTs: (1) Although this overview presents the option of the BJT to operate as an X-ray sensor in the medical diagnostic energy range, other studies are underway for such a transistor to be used in higher energy beams such radiotherapy and even higher energies like synchrotron light, for example; (2) As it was observed that the electrical state of the BJT systematically changes with the dose received by the device, some researchers have created an innovative technique for storing numerical data in a single transistor using X-rays [55]. Such a technique can bring a technological advance to increase the density of digital information per area in the memory integrated circuit. That is, with a high-resolution instrument for measuring the electrical state of the device, a 64-bit numerical value (which typically can use 64 or 128 transistors to store the value) can be stored in a single transistor, for example [56].

## 5. Conclusions

This overview demonstrated how the bipolar junction transistor can become an X-ray sensor in the energy range normally used in medical diagnosis. Some technical details regarding its internal semiconductor structure, electronics concepts, and radiation effects were discussed. The behavior of the irradiated BJT is presented based on these discussions and several references. Also, results were shown of how some BJT electrical parameters can vary after continuous use under X-ray beams. After the demonstrations of the BJT operating principle and the effects of radiation on some of its electrical parameters, a practical way of understanding how the transistor can operate as an X-ray sensor was presented. Finally, these studies actually open up a range of options for using the BJT in several other applications: radiotherapy, synchrotron light, innovative techniques for measuring some parameters in ionizing radiation beams, and its use in information technology as a digital memory cell based on the effect of X-ray beams.

## 6. Patents

1. Multimeter Electronic System for Measuring parameters of Diagnostic X-ray Equipment—“Sistema Eletrônico Multimedidor de Parâmetros de Equipamentos de Raios-X Diagnósticos”, Brazil Patent BR102015008361-0.
2. Electronic Memory System Using X-rays—“Sistema de Memória Eletrônica Usando Raios-X”, WIPO Patent PCT/BR2020/050387.

## Figures and Tables

**Table 1:** Some parameters of a BJT before, during and after an electrical stress (ES).

Parameter	*I_B_* (μA)	*h_FE_*	*I_C_* (mA)	Temperature (°C)
Before ES	100	120	12.0	21.5
During ES	12,500	24.3	303.8	115.4
After ES	100	123	12.3	21.4

**Table 2:** Main instruments used in this article.

Instrument	Model	Manufacturer	Lab.
Semiconductor analyzer	4200A-SCS	Keithley	1
Source-meter 1	6430	Keithley	1
Source-meter 2	6430	Keithley	1
Source-meter 3	2450	Keithley	2
Source-meter 4	EFF1705	Scients	2

**Table 3:** Influence of scattered radiation on the measurement of X-rays with the BJT.

Collimator Area (cm^2^)	~200	100	64	36	16	4	1	~0.2
*I_CX_* (nA)	1021.9	1009.4	1003.9	995.4	997.0	970.4	936.3	901.6

**Table 4:** Angular dependence of a ZTX851 BJT as a sensor in a diagnostic X-ray beam.

Rotation	Counterclockwise				Clockwise		
Angle	60°	45°	30°	0°	30°	45°	60°
*I_CX_* (nA)	951.8	988.4	1047.2	1020.5	1000.6	946.9	929.6

**Table 5:** Variation in *I_B_* to analyze its influence on the measurements of *I_CX_* and *I_BX_*.

BJT	NPN				PNP			
*I_B_* (nA)	*h_FE_*	*I_C_* (nA)	*I_CX_* (nA)	*I_BX_* (nA)	*h_FE_*	*I_C_* (nA)	*I_CX_* (nA)	*I_BX_* (nA)
5	100.0	500.2	704.0	7.0	101.9	509.4	483.7	4.7
10	100.6	1006.3	872.4	8.7	100.7	1007.4	650.6	6.5
20	100.7	2014.0	836.7	8.4	125.1	2501.8	601.1	4.8
30	100.0	3001.5	795.8	8.0	130.0	3899.1	555.4	4.3
40	100.2	4008.1	974.8	9.7	132.5	5301.9	646.0	4.9
50	103.3	5163.4	942.1	9.1	135.5	6774.1	705.3	5.2

**Table 6:** *I_BX_*(*J_BC_*) and *I_BX_*(*J_BE_*) results for a pair of BJTs (*β* ≈ 100) irradiated with 100 mAs.

BJT	X-ray Tube Potential (kV)	77	102	125
npn *β* ≈ 100	Measured *I_BX_*(*J_BC_*) (nA)	5.2	8.3	11.0
	Measured *I_CX_* (nA)	569.5	922.3	1229.1
	Estimated *I_BX_*(*J_BE_*) (nA)	0.5	0.9	1.3
pnp *β* ≈ 100	Measured *I_BX_*(*J_BC_*) (nA)	2.9	4.9	6.3
	Measured *I_CX_* (nA)	396.7	652.3	874.5
	Estimated *I_BX_*(*J_BE_*) (nA)	1.1	1.6	2.4

**Table 7:** *I_BX_*(*J_BC_*) and *I_BX_*(*J_BE_*) results for a pair of BJTs (*β* ≈ 100) irradiated with 200 mAs.

BJT	X-ray Tube Potential (kV)	77	102	125
npn *β* ≈ 100	Measured *I_BX_*(*J_BC_*) (nA)	9.9	16.2	21.9
	Measured *I_CX_* (nA)	1099.9	1770.8	2356.1
	Estimated *I_BX_*(*J_BE_*) (nA)	1.1	1.5	1.7
pnp *β* ≈ 100	Measured *I_BX_*(*J_BC_*) (nA)	5.6	9.3	12.4
	Measured *I_CX_* (nA)	799.3	1282.1	1722.7
	Estimated *I_BX_*(*J_BE_*) (nA)	2.4	3.5	4.8

**Table 8:** Variation of *h_FE_* and *r_o_* parameters of a pair of BJTs after irradiation (|*V_CE_*| = 25 V).

BJT Type	TIP41 (npn)		TIP42 (pnp)	
Dose (Gy)	*h_FE_*	*r_o_* (kΩ)	*h_FE_*	*r_o_* (kΩ)
0	35.6	76.2	25.6	54.2
20	30.7	90.8	20.9	59.7
40	26.1	111.5	17.5	70.5
60	22.1	135.5	14.3	77.9
80	18.5	171.3	11.9	91.4
100	15.6	212.0	10.4	102.6
